# Synthesis of TiO_2_-(B) Nanobelts for Acetone Sensing

**DOI:** 10.3390/s23198322

**Published:** 2023-10-08

**Authors:** Gayan W. C. Kumarage, Shasika A. Panamaldeniya, Dileepa C. Maddumage, Abderrahim Moumen, Valentin A. Maraloiu, Catalina G. Mihalcea, Raluca F. Negrea, Buddhika S. Dassanayake, Nanda Gunawardhana, Dario Zappa, Vardan Galstyan, Elisabetta Comini

**Affiliations:** 1 SENSOR Lab, Department of Information Engineering, University of Brescia, 25133 Brescia, Italy or chathurangakumarage@gmail.com (G.W.C.K.);; 2 Department of Physics and Electronics, Faculty of Science, University of Kelaniya, Kelaniya 11600, Sri Lanka; 3 Postgraduate Institute of Science, University of Peradeniya, Peradeniya 20400, Sri Lanka; 4 Department of Physics, Faculty of Science, University of Peradeniya, Peradeniya 20400, Sri Lanka; 5 Laboratory of Atomic Structures and Defects in Advanced Materials, National Institute of Materials Physics, Atomistilor str. 405 A, 077125 Magurele, Romania; maraloiu@infim.ro (V.A.M.);; 6 Research and International Affairs, Sri Lanka Technological Campus, Padukka 10500, Sri Lanka

**Keywords:** TiO_2_ nanobelts, hydrothermal synthesis, chemical sensing, acetone detection

## Abstract

Titanium dioxide nanobelts were prepared via the alkali-hydrothermal method for application in chemical gas sensing. The formation process of TiO_2_-(B) nanobelts and their sensing properties were investigated in detail. FE-SEM was used to study the surface of the obtained structures. The TEM and XRD analyses show that the prepared TiO_2_ nanobelts are in the monoclinic phase. Furthermore, TEM shows the formation of porous-like morphology due to crystal defects in the TiO_2_-(B) nanobelts. The gas-sensing performance of the structure toward various concentrations of hydrogen, ethanol, acetone, nitrogen dioxide, and methane gases was studied at a temperature range between 100 and 500 °C. The fabricated sensor shows a high response toward acetone at a relatively low working temperature (150 °C), which is important for the development of low-power-consumption functional devices. Moreover, the obtained results indicate that monoclinic TiO_2_-B is a promising material for applications in chemo-resistive gas detectors.

## 1. Introduction

Metal oxide-based chemical gas sensors are now regarded as excellent candidates for environmental monitoring due to their good sensitivity, fast response, and recovery times toward different analytes [1,2,3,4,5,6,7]. Moreover, research studies are progressing to employing metal oxides at their optimum gas-sensing performances and develop low-cost, portable, and stable chemo-resistive sensors. Among the existing technologies of metal oxides, thick films, thin films, nanocrystals, and one-dimensional (1D) structures are widely investigated owing to their superior surface-to-volume ratio, stability, and low production cost, together with excellent analyte-sensing performances [8,9,10,11,12]. Furthermore, 1D SnO_2_, ZnO_2_, and TiO_2_ materials are widely studied in the field of chemo-resistive sensors, accounting for over 78% (SnO_2_ > 34%, ZnO_2_ > 31%, and TiO_2_ > 12%) of all reported 1D metal oxide chemical gas sensors, to the best of the authors’ knowledge [13,14].

**Figure 1:**
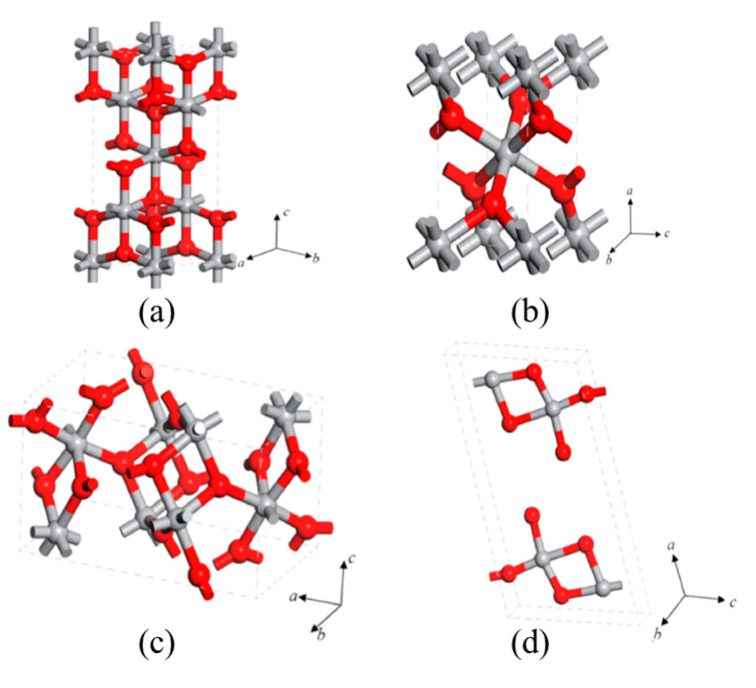
The crystal structures of TiO_2_: (**a**) anatase, (**b**) rutile, (**c**) brookite, and (**d**) monoclinic. Reprinted with permission from [22].

TiO_2_ is one of the interesting transition-metal oxides due to its biocompatibility, stability, abundance, and low cost of production [15,16,17,18,19,20,21]. The crystallite phases of TiO_2_, i.e., anatase, rutile, brookite, and monoclinic, are shown in Figure 1a–d [22]. The chains of TiO_6_ octahedra are the building blocks that make up the tetragonal lattice structures of TiO_2_. In an anatase structure, zigzag chains of TiO_6_ octahedra are linked to each other with four edge-shared bonding (faces sharing, Figure 1a), and, in the rutile phase, two opposite edges of each octahedron link at a corner of an oxygen atom, forming linear chains of octahedra at each corner (Figure 1b). Consequently, rutile is the most stable crystal structure in the bulk material. However, brookite structures (Figure 1c) are also commonly available based on specific growth techniques and mechanisms [23]. Thus, investigating novel approaches for the synthesis of TiO_2_ nanostructures including simple fabrication technologies is still demanding. Hence, several growth techniques, for instance, atomic layer deposition [24], electrochemical anodization [25], hydrothermal method [26], chemical vapor deposition (CVD) [27], and electrospinning [28], have been employed to fabricate low-dimensional TiO_2_ structures for gas-sensing applications [29,30].

Besides these three crystal phases, another crystal phase, TiO_2_-(B) (monoclinic, Figure 1d), is also available based on synthesis parameters. TiO_2_-(B) possesses a layer structure, resulting in low density, high specific capacity, and more open structural frameworks compared to other phases [30,31]. The various TiO_2_ crystal phases react with interacting gases in distinctive ways, as is well known [18]. Due to their molecular structure, bond length, and reactive groups, crystal phases are sensitive to certain gases [32]. For instance, rutile TiO_2_ nanorods are sensitive to isopropanol [33] and anatase TiO_2_ nanowires are sensitive to NO_2_ [34]. As a result, it is crucial to research the various TiO_2_ crystal phases for gas sensing. However, monoclinic TiO_2_ is very rarely reported in the literature because of synthesis difficulties. Pioneering work on TiO_2_-(B) was reported by Marchand et al. in 1980, which was prepared using the hydrothermal method, one of the simplest, low-cost, and high-yield processes [35]. Subsequently, several interesting research works have been reported on the preparation of TiO_2_-(B), showing high catalytic and electrochemical properties [36,37,38,39].

Moreover, the hydrothermal method is a well-known process for preparing metal oxide nanomaterials with various morphologies by simply varying the pH value of the solution and the growth temperature. However, some highly toxic chemicals are still involved in these growth methods to achieve high-quality nanostructures. Therefore, we investigated the potential of replacing highly toxic compounds with low- or moderately toxic chemicals to grow TiO_2_-(B) nanobelts via low-cost hydrothermal techniques. Herein, harmful hydrochloric acid (HCl), which is usually used in the protonation phase, was replaced by acetic acid (CH_3_COOH) for the synthesis of TiO_2_-(B). Furthermore, the morphological, structural, and compositional characteristics of the material were investigated. Additionally, the gas-sensing properties of TiO_2_-(B) were determined in order to employ the prepared material in low-power-consumption chemiresistive gas sensors.

## 2. Materials and Methods

### 2.1. Growth of TiO_2_-B Nanobelts

The alkali-hydrothermal process was employed for the synthesis of TiO_2_ nanobelts. First, sodium titanate hydrated (Na_2_Ti_3_O_7_.mH_2_O) was synthesized using the hydrothermal method. A quantity of 1 g of titanium dioxide (TiO_2_, powder, 21 nm primary particle size, ≥99.5%, Aldrich) was mixed with 70 mL of 10.0 mol dm^−^^3^ sodium hydroxide (NaOH, 98%, Loba Chemie, Mumbai, India) aqueous solution and mechanically stirred for 30 min, followed by 15 min of ultrasonication. The stirring and sonication processes were repeated six times following each other. Later, the obtained suspension was transferred into a 100 mL Teflon-lined stainless-steel autoclave and thermally treated for 48 h at five different temperatures: 120 °C, 135 °C, 150 °C, 175 °C, and 200 °C. After that, the material was cooled down to room temperature. The resultant white slurry (Na_2_Ti_3_O_7_) was washed thoroughly with deionized water, followed by a filtration process until the pH of the washing solution reached the value of 7. Subsequently, the wet slurry was immersed in 1 mol L^−^^3^ acetic acid (99.5%, DAEJUNG, Sihung, Republic of Korea) aqueous solution for 24 h to prepare the protonated titanate form (H_2_Ti_3_O_7_). The prepared H_2_Ti_3_O_7_ was washed thoroughly with distilled water and underwent filtration until the washing solution became pH neutral. Later, the obtained H_2_Ti_3_O_7_ was dried at 80 °C for 24 h, and then calcinated at 500 °C for 3 h.

The basic chemical routing of the TiO_2_ nanobelt growth can be written as follows [40]:

The reaction starts with the dissolving of TiO_2_, in the presence of NaOH, as

3TiO_2_ + 2NaOH → Na_2_Ti_3_O_7_ + H_2_O (1)

During the CH_3_COOH washing, the Na_2_Ti_3_O_7_ nanobelt is known to convert as follows due to the ion exchange:

Na_2_Ti_3_O_7_ + 2CH_3_COOH → H_2_Ti_3_O_7_ + 2CH_3_COO^−^Na^+^ (2)

In the calcination process, H_2_Ti_3_O_7_ will convert to TiO_2_ as follows:

H_2_Ti_3_O_7_ → 3TiO_2_ + H_2_O (3)

### 2.2. Characterization

The morphological investigations were carried out using a field-emission scanning electron microscope (FE-SEM) TESCAN (Brno, Czech Republic) (MIRA-3) at a voltage of 5 kV. Further, the obtained nanostructures were investigated by means of a JEOL JEM ARM 200F analytical transmission electron microscope (TEM) operated at 200 kV, equipped with an EDS detector to acquire Energy-Dispersive X-ray (EDS) spectra or maps for elemental investigation. X-ray diffraction spectroscopy (XRD) was performed in the range of 20–80 degrees using an Empyrean diffractometer (PANalytical, Almelo, The Netherlands) equipped with a Cu-kα_1_ (λ = 1.5406 Å) tube operating at 40 kV to 40 mA. Raman spectra were measured using a fiber-coupled confocal optical microscope (HORIBA, XploRA Nano) at 100× magnification. The spectra were recorded in the wavelength range of 200–1200 cm^−1^ using a red laser source (638 nm).

### 2.3. Fabrication of Sensors

The drop-casting method was used to fabricate the conductometric sensing devices and several sensors were fabricated by varying the number of droplets, as shown in Table 1. The process can be explained in brief as follows: Alumina substrates (2 mm × 2 mm, Kyocera, Kanazawa, Japan, 99.9%) were ultrasonically cleaned for 20 min in an acetone bath, followed by drying with synthetic air. Next, TiW (10/90 wt.%) adhesion layer (~90 nm) was sputtered on top of the alumina substrates via DC magnetron sputtering at 75 W argon plasma, 7 (standard cubic centimeters per minute) argon flow, 5.0 mTorr pressure, and 300 °C. Then, Pt interdigitated electrodes (IDE, 1 μm) were deposited on the adhesion layer using the same DC magnetron sputtering conditions [41]. Additionally, a platinum heater was fabricated on the opponent side of the alumina substrates to investigate the performance of the sensor at different operating temperatures (100–500 °C). Meanwhile, the Al_2_O_3_ substrates with IDE and heater were soldered to the TO-39 package with gold wires.

**Figure 2:**
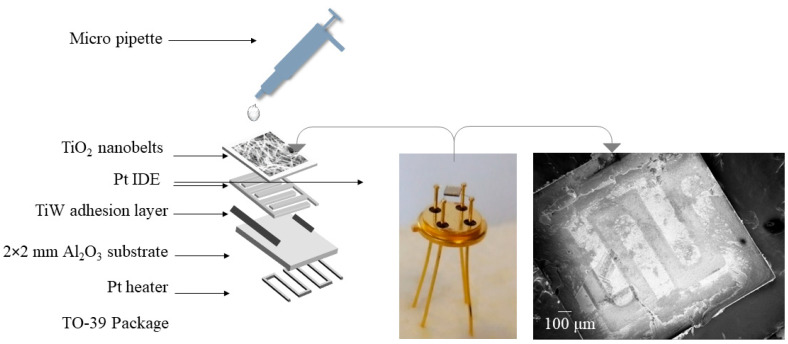
Schematic of the fabricated conductometric chemical gas sensor and the electrodes.

Later, the dispersed TiO_2_ nanobelts (5 mg) in absolute ethanol (5 mL) were drop-casted on the top of the soldered device at room temperature. Figure 2 shows the schematic of the prepared sensors.

### 2.4. Gas Testing Measurements

All the fabricated sensors were thermally stabilized at 400 °C for 48 h before mounting into a gas-testing chamber. The conductometric response of the fabricated sensors was tested in a stainless-steel gas chamber having a volume of 1 L, which was mounted inside a climatic chamber (Angelantoni, Perugia, Italy, model MTC 120). The temperature was set to 20 °C inside the climatic chamber, and 40% of relative humidity (RH) was maintained inside the gas chamber. To create humid air, dry air flowed through a Drechsel bottle operating at a water bath of 25 °C. Subsequently, the sensors were mounted in the gas chamber to investigate their response toward H_2_, C_2_H_5_OH, C_3_H_6_O, NO_2_, and CH_4_ with a fixed voltage of 1 V. The gas-sensing dynamic was recorded for the period of 140 min for each concentration (30 min injection followed by 45 min recovery). The response of sensors is defined as ∆G/G (S) = (G_g_ − G_a_)/G_a_ or (G_a_ − G_g_)/G_g_ for reducing and oxidizing gases, respectively, where G_a_ is the conductance of the sensor in synthetic air while G_g_ is the conductance of the sensor in the presence of analyte gas. The gas-sensing measurements were carried out at a gas flow rate of 200 sccm.

## 3. Results

**Figure 3:**
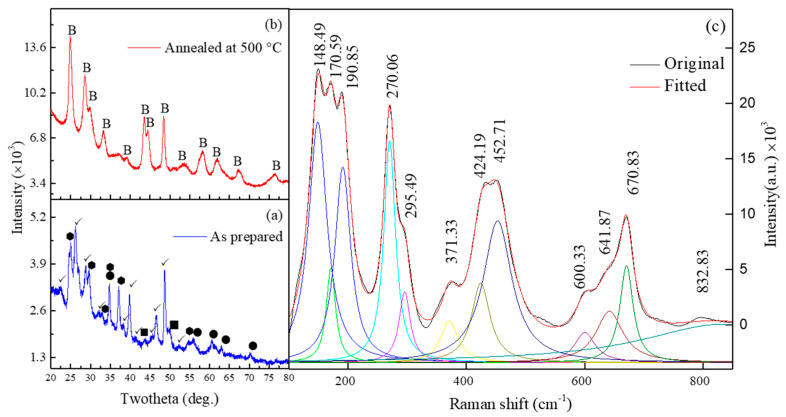
XRD patterns of samples: (**a**) intermediate H_2_Ti_3_O_7_, (**b**) TiO_2_-(B), and (**c**) Raman spectra of the prepared H_2_Ti_3_O_7_ at 200 °C before calcination. B—TiO_2_-(B); ✓—H_2_Ti_3_O_7_; ●—Na_2_Ti_3_O_7_; ■—Na_2_Ti_4_O_9_; 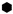
—Na_2_Ti_9_O_19_. The oridinal Ramana spectra is shown in black and the fitted Ramana spectra is shown in Red. Whle other are the peak fitting corresponding to fitted spectra (Red).

To study the formation and crystal structure of the prepared TiO_2_, X-ray diffraction (XRD) analysis was employed. Figure 3a clearly shows the formation of intermediate H_2_Ti_3_O_7_ and residual sodium titanate (JCPDS 41-0192, JCPDS 98-008-2059). However, these H_2_Ti_3_O_7_ and residual sodium titanate have been completely demolished upon washing and annealing (Figure 3b). Further, H_2_Ti_3_O_7_ is the most important intermediate state, which results in the formation of nanobelt-like morphology in the final product ascribed to the dissolution–recrystallization process [42]. After OH^−^ ions in the NaOH solution have gradually diffused into the initial 3D anatase TiO_2_, the Ti–O–Ti bonds are dissolved, leading to the exfoliation of single-layered sheets (nanosheets) composed of TiO_6_ octahedra. This dissolution is the main root for the presence of characteristic stretching vibrational peaks in the Raman spectrum at 280 cm^−^^1^ (Ti–O–Na), 372 cm^−^^1^ (Ti–O–Ti), and 670.8 cm^−^^1^ (TiO_6_) [38,39], as shown in Figure 3c.

**Figure 4:**
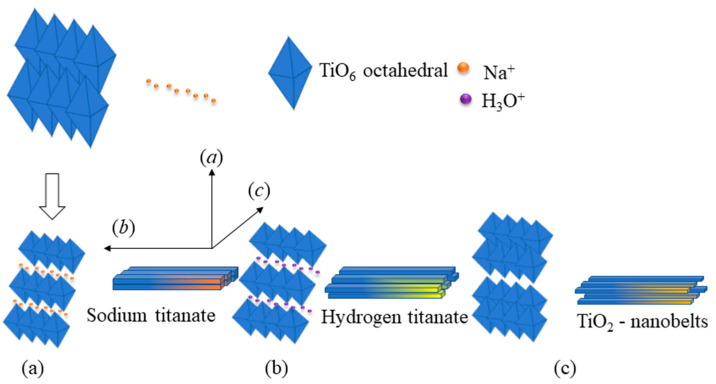
Schematic formation of (**a**) sodium titanate (Na_2_Ti_3_O_7_) nanosheets, (**b**) hydrogen titanate (H_2_Ti_3_O_7_) nanobelts, and (**c**) TiO_2_ nanobelts.

**Figure 5:**
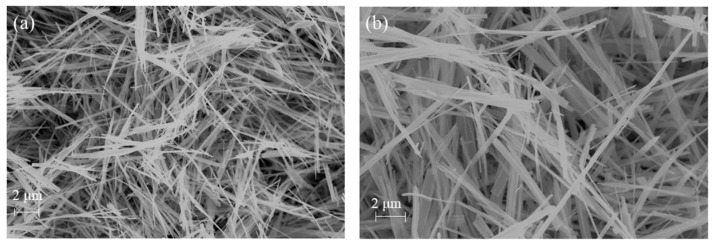
FE-SEM of (**a**) H_2_Ti_3_O_7_ prepared in the autoclave at 200 °C, and (**b**) TiO_2_-(B) annealed at 500 °C.

Meanwhile, Na^+^ ions intercalate in the interlayer space between TiO_6_ sheets to neutralize the negative charge of the formed TiO_6_ sheets, as shown in Figure 4a. This step controls the Na/Ti ratio of the final titanate product and forms intermediate sodium titanate (Na_2_Ti_3_O_7_), as described in Equation (1) [28]. However, such dissolution and exfoliation processes are relatively easier for small anatase precursors yielding titanate sheet units. These sodium titanate sheets copy the epitaxial crystal growth along the c-axis, resulting in titanate sheet units growing into a sheet-like structure (Figure 5a) [43]. Next, Na_2_Ti_3_O_7_ nanosheets are transformed into H_2_Ti_3_O_7_ nanobelts when the ion-exchange process is employed (Equation (2) and Figure 4b). Accordingly, the formation of nanobelt-like structure is ascribed to the splitting of nanosheets to release the excess strong stress upon the replacement of Na^+^ by larger H_3_O^+^ cations when forming H_2_Ti_3_O_7_ [44]. Finally, TiO_2_-B nanobelts are obtained by annealing the H_2_Ti_3_O_7_ at 500 °C, as presented in Equation (3) and Figure 4c.

The diffraction peaks in Figure 3b at the two-theta values of 24.98°, 28.59°, 29.79°, 33.41°, 39.47°, 43.48°, 44.61°, 48.66°, 52.89°, 58.47°, 62.38°, 67.27°, and 76.69° are, respectively, assigned to the reflections of the (110), (002), (40-1), (31-1), (31-2), (003), (60-2), (020), (113), (71-1), (31--), (02-3), and (712) planes of the monoclinic phase (JCPDs: 98-004-7691, space group C12/m1) TiO_2_-B.

Figure S1a–f show FE-SEM images of the intermediate H_2_Ti_3_O_7_ structures, which were synthesized by dissolving anatase TiO_2_ powder (particle diameter 20–25 nm, P21, Figure S1a) with NaOH solution under hydrothermal conditions and using the ion-exchange process with acetic acid. Figure S1b shows that the hydrothermal treatment at 120 °C results in the transformation of anatase particles into a compact and thick flake-like morphology. Further, at 135 °C, anatase particles were transferred into large aggregated particles (Figure S1c). At 150 °C, these P21 particles aggregated and formed a compact film-like morphology (Figure S1d). However, P21 particles were completely transformed into belt-like shapes (Figure S1e–f) when the hydrothermal treatment was employed at 175 °C and 200 °C. Furthermore, the obtained nanobelts at 200 °C showed a compact and low aspect ratio. Accordingly, further investigation was carried out on the sample that was thermally treated at 200 °C.

**Figure 6:**
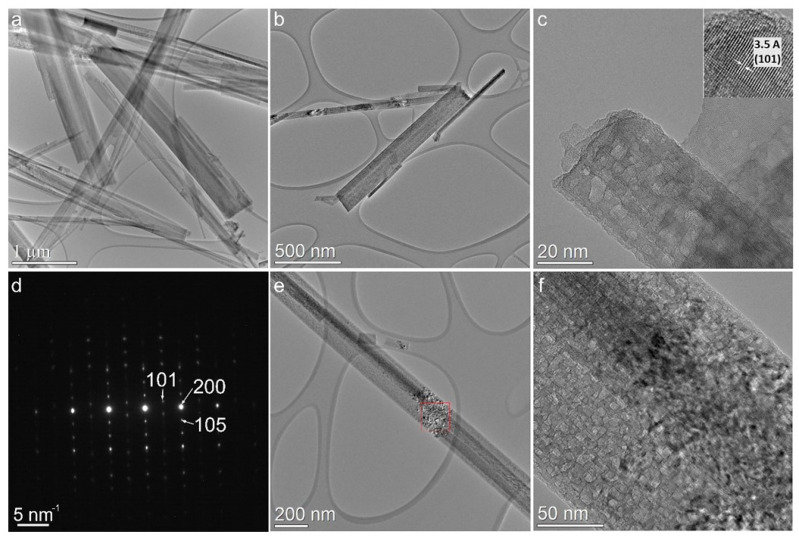
CTEM images: (**a**) H_2_Ti_3_O_7_, (**b**) TiO_2_-(B), and (**e**) formation of pore-like structure in TiO_2_-(B); (**c**) HRTEM image of TiO_2_-(B) with insert showing (101) planes; and (**d**) SAED of TiO_2_-(B); (**f**) magnified image of (**c**).

The conventional TEM (CTEM) image in Figure 6a shows a one-dimensional nanobelt structure in the obtained intermediate H_2_Ti_3_O_7_ to a very long extent in the range of ten micrometers. Figure 6b shows a CTEM image of TiO_2_ nanobelts, which were annealed at 500 °C. The HRTEM image (Figure 6c), with its insert clearly showing (101) planes that are characteristic of anatase structure, demonstrates that the nanobelts are well crystallized. Figure 6d shows the selected-area electron diffraction (SAED) of such a nanobelt, which confirms the presence of (101), (200), and (105) crystallographic planes of TiO_2_-(B) (CIF number 9009086) corresponding to the lattice parameters a = b = 3.785 Å, c = 9.514 Å, and *α* = *β* = *γ* = 90°. Also, Figure 6d clearly shows the formation of nanobelts in the direction of the c-axis. Furthermore, Figure 6e,f show the presence of a “porous-like” morphology in some regions of the prepared TiO_2_-(B) due to the large density of structural defects in specific areas along the nanobelts.

**Figure 7:**
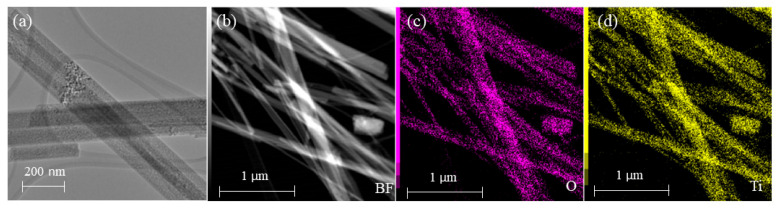
(**a**) CTEM of several overlapping TiO_2_-(B) nanobelts; EDS analysis of TiO_2_-(B) nanobelts. The maps show the (**c**) O and (**d**) Ti distribution on the nanobelts corresponding to the HAADF image of (**b**).

Energy-Dispersive X-ray (EDS) spectra on the TEM grid were carried out to study the elemental distributions on the surface of TiO_2_-(B) nanobelts. The EDS maps (Figure 7a–c) indicate the presence of constituent elements (Ti and O) on the surface of TiO_2_-(B) nanobelts. Remarkably, Figure 7a–d also show some overlapping nanobelts, which can significantly enhance the response of the sensor when employed in gas sensing [45].

### Gas-Sensing Performance

**Figure 8:**
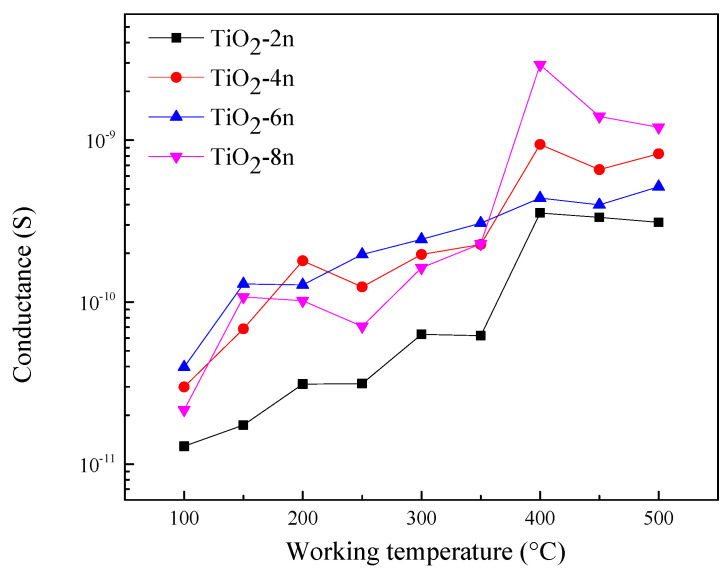
Electrical conductance variation of the fabricated sensors at different working temperatures under 40 RH% conditions.

Figure 8 shows the conductance-temperature behavior of the fabricated TiO_2_-(B) sensors. The conductance was found to increase with increasing operating temperature of the sensors, which is ascribed to the semiconducting characteristics of the prepared TiO_2_-(B) material. However, the sensors fabricated with eight drops (TiO_2_-8n) show the highest conductivity at the operating temperature of 400 °C.

Preliminary gas-sensing studies were carried out at different working temperatures. The highest conductivity was observed at 400 °C. Interestingly, the sensors (TiO_2_-8n) demonstrated the highest response toward acetone compared to other TiO_2_ sensors. Therefore, we further analyzed the sensing properties of the TiO_2_-8n sensor.

We investigated the gas-sensing properties of TiO_2_-8n devices toward hydrogen (H_2_), methane (CH_4_), nitrogen dioxide (NO_2_), ethanol (C_2_H_5_OH), and acetone (C_3_H_6_O) at the temperature range of 100–500 °C (step, 50 °C). The electrical conductance of the sensors increases to a maximum value once reducing gases are injected and returns to their initial value when the gas flow is not present in the chamber. This is a typical n-type semiconducting behavior, which is in agreement with the results reported in the literature [46,47]. Usually, working temperature affects the gas interaction with the sensor up to a certain catalytic temperature and then hinders the interaction, thereby modulating the response value. Therefore, metal oxide gas sensors have different response values depending on their working temperature. Thus, investigating the optimum working temperature is significantly important with respect to the performance of the sensors.

**Figure 9:**
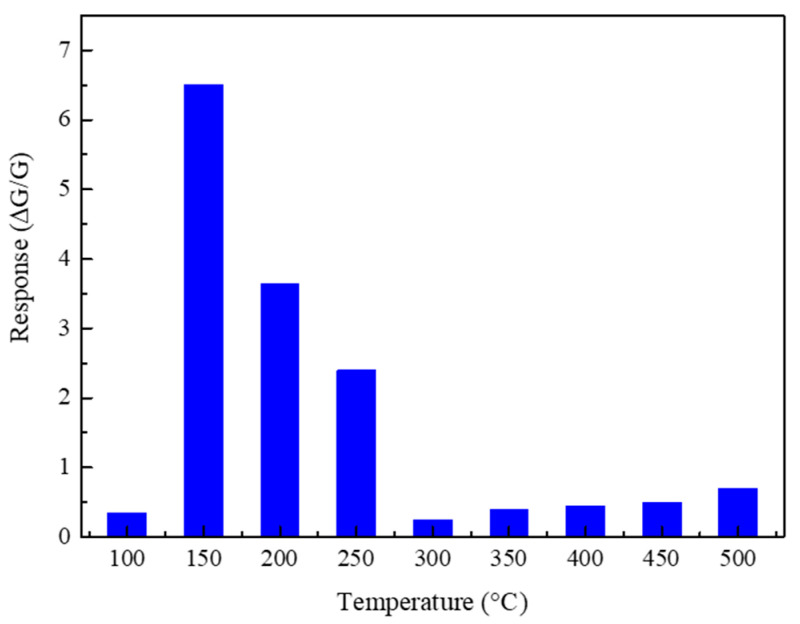
Response of the TiO_2_ nanobelts (TiO_2_-8n) to 10 ppm C_3_H_6_O at different working temperatures in 40% RH conditions.

**Figure 10:**
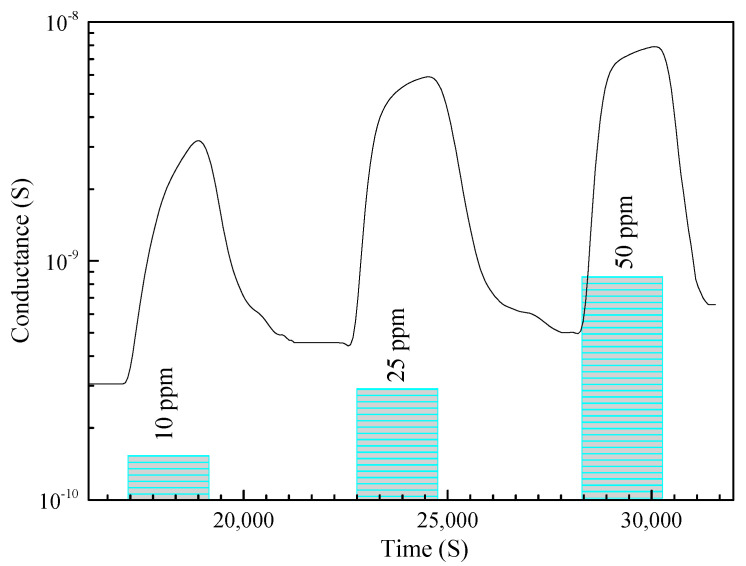
Dynamic response–recovery plot of the TiO_2_-8 sensor toward 10, 25, and 50 ppm of C_3_H_6_O at the working temperature of 150 °C.

The response values of the TiO_2_-8n sensor toward 10 ppm of C_3_H_6_O at different working temperatures are shown in Figure 9. The sensor exhibited the highest response at the working temperature of 150 °C. Figure 10 shows the dynamic response plot of the TiO_2_-8n sensor to different concentrations of C_3_H_6_O at the operating temperature of 150 °C. TiO_2_-based gas sensors mostly operate at elevated working temperatures (>200 °C). So, the relatively low working temperature of the fabricated sensors can be due to the high catalytic property of TiO_2_-(B), which is also evidenced in the conductance variation of the sensors [48]. Also, porous regions in the nanobelts encourage the oxygen adsorption/desorption mechanism [44]. Accordingly, the porous-like region of the prepared nanobelts can potentially be one of the reasons for the high response at low working temperatures [49].

**Figure 11:**
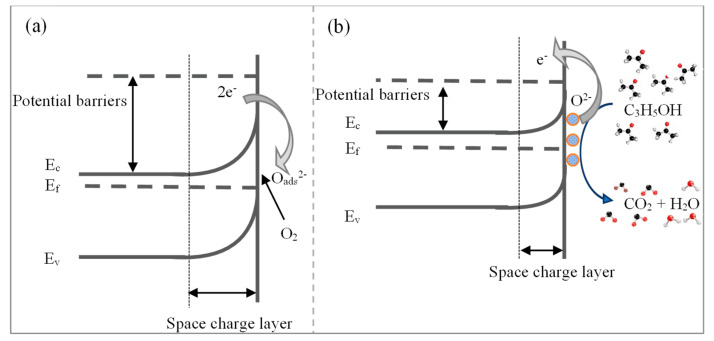
(**a**) Electrical band bending due to the adsorption of oxygen species to the TiO_2_ nanobelt surface (O_2_^−^_ads_, O^−^_ads_, O^2−^_ads_), and (**b**) reduction in the space charge region resulting in a decrement in electrical resistance due to the interaction between acetone molecules and TiO_2_ surface. E_c_ is the conduction band, E_v_ is the valence band, and E_f_ is the Fermi level.

In general, the gas-sensing mechanism of TiO_2_-(B) nanobelts is acerbated by surface reactions and induced carrier charge transfer due to the adsorption of analyte gas molecules. Furthermore, the sensing mechanism is based on two main interactions known as oxygen adsorption and C_3_H_6_O interaction on the surface of TiO_2_ nanobelts. The sensing mechanism is related to the adsorption of oxygen onto the surface of TiO_2_-(B) nanobelts. Usually, the adsorption of oxygen molecules occurs on the exposed surface of the MOX surface [50]. Consequently, electron transfers from the conduction band of TiO_2_ to oxygen molecules result in the formation of oxygen species at the surface. Typically, three types of oxygen species ($O_{2}^{-}$, O^−^, O^2−^) are formed depending on the temperature. The formation of oxygen species is shown in Equations (4)–(7). Furthermore, the extraction of electrons by oxygen molecules leads to an increase in the depletion layer width. Accordingly, resistance increases, as shown in Figure 11a [35].

O_2(gas)_ ↔ O_2(ads)_ (4)

O_2(ads)_+ e^−^ ↔ O_2_^−^_(ads)_ (5)

(6) $${O_{2}^{-}}_{\left(\mathrm{ads}\right)}+e^{-}↔{2O^{-}}_{\left(\mathrm{ads}\right)}$$

O^−^_(ads)_ + e^−^ ↔ O^2−^_(ads)_ (7)

Once the sensor is exposed to acetone, it interacts with the adsorbed oxygen molecular species. The interaction with O_2_^−^ is interesting to discuss here because the optimum working temperature is 150 °C. This interaction leads to the decomposition of C_3_H_6_O into carbon dioxide (CO_2_) and water (H_2_O), as shown in Equations (8) and (9) [50]. Thus, the released electrons (e^−^) reduce the depletion layer, hence lowering the resistance of the sensor, as shown in Figure 8b.

CH_3_COCH_3(gas)_ ↔ CH_3_COCH_3(ads)_ (8)

CH_3_COCH_3(ads)_ + 8O^−^_(ads)_ → 3CO_2(gas)_ + 3H_2_O_(gas)_ + 8e^−^ (9)

**Figure 12:**
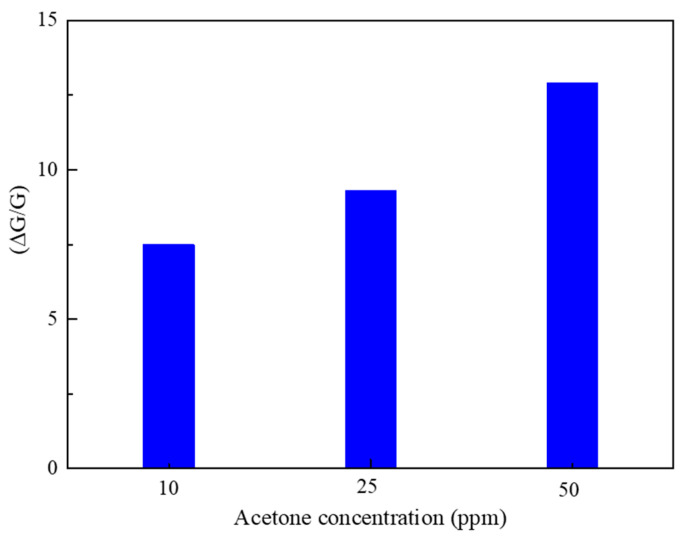
Variation in the sensor’s response toward different concentrations of C_3_H_6_O at the working temperature of 150 °C.

The sensing characteristics of the structures indicate that their response is enhanced by increasing the concentration of C_3_H_6_O (Figure 12). Conversely, a higher gas concentration results in a higher number of surface interactions, thus resulting in a higher response [51]. The porosity of the nanostructures is one of the possible reasons for the not-too-slow gas adsorption and diffusion within the sensor, resulting in the estimated response and recovery times of 348 s and 600 s at 40 RH% toward 10 ppm C_3_H_6_O. The estimated response and recovery times are shown in Table 2.

**Figure 13:**
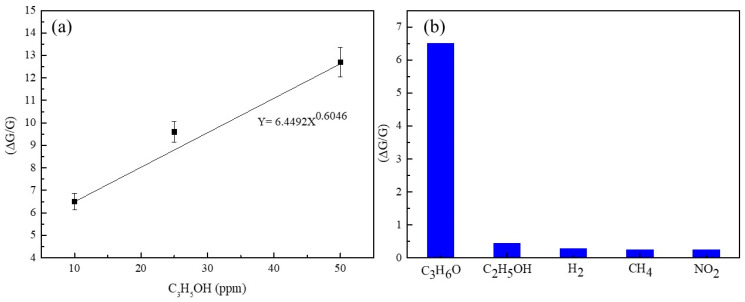
(**a**) The plot of response vs. concentration value of the sensor (TiO_2_-8n) toward C_3_H_6_O at 150 °C. (**b**) Selectivity of the TiO_2_-8n sensor toward the tested gases (10 ppm C_3_H_6_O, 10 ppm C_2_H_5_OH, 100 ppm H_2_, 100 ppm CH_4_, and 1 ppm NO_2_) in 40 RH% humidity air at the operating temperature of 150 °C.

A gas-sensing material with a lower detection limit may have a good response to a smaller quantity of targeted gas, which ultimately helps to prevent serious accidents in a polluted environment. Hence, the power fitting (y = 6.4492x^0.6046^) between response and concentration is a significantly important characteristic (Figure 13a), in which the gradient describes the sensitivity and the intercepts show the detection limit. As shown in Figure 13b, the fabricated sensors have a relatively better response with a detection limit of ~0.05 ppm (∆G/G = 1 on power fitting). Concerning the practical application of the sensors, reproducibility and stability in response are also considered essential parameters.

Hence, the reproducibility and stability of TiO_2_-(B) nanobelts toward C_2_H_6_O were studied and reported herein. Figure S2 shows the reproducibility of the sensor toward three consecutive cycles of 50 ppm C_3_H_6_O at 150 °C and 40 RH%. Almost the same response value is achieved upon successive injections of C_3_H_6_O. Figure S3 shows the stability plot for two weeks toward 50 ppm C_3_H_6_O at 150 °C in 40 RH%. Figure S3 clearly shows the excellent performances of the sensors’ response during the tested period. Figure S4 displays the response values of the TiO_2_-8 sensor toward different gases at different working temperatures. Furthermore, Figure 13b depicts the selectivity of the TiO_2_-8 sensor toward the 10 ppm C_3_H_6_O, 10 ppm C_2_H_5_OH, 100 ppm H_2_, 100 ppm CH_4_, and 1 ppm NO_2_ at the operating temperature of 150 °C.

Thus, the observed C_3_H_6_O-sensing performances, for instance, response, selectivity, response/recovery times, reproducibility, and stability, of the TiO_2_-B nanobelts make them a potential candidate for selective sensing of C_3_H_6_O in a variety of applications when comparing the literature survey shown in Table 3.

## 4. Conclusions

Monoclinic TiO_2_ nanobelts were successfully prepared using the hydrothermal method. These studies indicated that the employment of acetic acid was successful in the protonation phase of TiO_2_-(B). The fabricated TiO_2_ gas sensors showed good gas-sensing performance. The highest response (∆G/G) was 6.5 toward 10 ppm of acetone at the working temperature of 150 °C. Moreover, the sensors demonstrated good selectivity toward acetone compared to ethanol, hydrogen, methane, and nitrogen dioxide. Thus, the fabricated monoclinic TiO_2_-B nanobelts are promising candidates for developing functional gas-sensing devices that work at relatively low power consumption. Additionally, the potential of lower detection limit and high selectivity toward acetone could make it interesting to employ the synthesized monoclinic TiO_2_ nanobelts in chemiresistive gas sensor applications.

## Figures and Tables

**Table 1:** Nomenclature of the fabricated conductometric chemical sensors.

Number of Drops	Nomenclature
2	TiO_2_-2n
4	TiO_2_-4n
6	TiO_2_-6n
8	TiO_2_-8n

**Table 2:** Sensors’ performance to different concentrations of C_3_H_6_O.

C_3_H_6_O (ppm)	40 RH%			
	Response	Response	Response Time (S)	Recovery Time (S)
10	1.3	6.5	378	600
25	2.1	9.6	348	960
50	4.4	12.7	324	1320

**Table 3:** Comparison of gas-sensing parameters of TiO_2_-B nanobelt sensor with other reported TiO_2_-based C_3_H_6_O sensors.

Material	Synthesis Route	Working Tempt. (°C)	Response (R_a_/R_g_)	C_3_H_6_O (ppm)	Res. Time (T_res_) (s)	Rec. Time (T_rec_) (s)	The Lowest Detection Limit (ppm)	Ref.
TiO_2_ porous NPs	Hydrothermal	275	13.9	100	11	14	-	[52]
TiO_2_ NPs	Matrix-assisted pulsed laser deposition	400	6	100	240	-	20	[53]
Nanoporous TiO_2_	Hydrothermal	370	25.97	500	13	8	20	[54]
Ag-TiO_2_ nanobelts	Hydrothermal	260	28.25	500	6	8	0.8	[55]
TiO_2_ nanorods	Electrospun	500	13	300	12	6	-	[56]
TiO_2_-B nanorods	Hydrothermal	320	2.3	100	3	180	-	[57]
TiO_2_-B nanobelts	Hydrothermal	150	12.7	50	324	1320	0.7	This work

## Data Availability

The data presented in this study are available on request from the corresponding author.

## Supplementary Materials

The following supporting information can be downloaded at https://www.mdpi.com/article/10.3390/s23198322/s1. Figure S1: FE-SEM images of the obtained morphology at (a) starting anatase TiO2 powder, (b) hydrothermal treatment at 120 °C, (c) hydrothermal treatment at 135 °C, (d) hydrothermal treatment at 125 °C, (e) hydrothermal treatment at 175 °C, and (f) hydrothermal treatment at 200 °C. Figure S2: Reproducibility study of TiO_2_-B nanobelt sensor toward 50 ppm C_3_H_6_O at 150 °C under 40 RH% condition. Figure S3: Stability study of TiO_2_-B nanobelt sensor toward 50 ppm C_3_H_6_O at 150 °C under both dry and 40 RH% conditions. Figure S4: Response values of the sensor toward 500, 100, 10, 10, and 10 ppm of CH_4_, H_2_, NO_2_, C_2_H_5_OH, and C_3_H_5_OH.
