# Supplementary material for: Synthesis of TiO2-(B) Nanobelts for Acetone Sensing
Source: Sensors (Basel). 2023 Oct 8;23(19):8322. doi: 10.3390/s23198322 (PMC10575087; doi:10.3390/s23198322)
Supplement: Supplementary file 1 [file sensors-23-08322-s001.zip › sensors-2616018-supplementary.pdf]

Synthesis of TiO2-(B) Nanobelts for Acetone Sensing

Gayan W. C. Kumarage [1,2], Shasika A. Panamaldeniya [3,4], Dileepa C. Maddumage [3,4], Abderrahim Moumen [1], Valentin A. Maraloiu [5], Catalina G. Mihalcea [5], Raluca F. Negrea [5], Buddhika S. Dassanayake [4], Nanda Gunawardhana [6], Dario Zappa [1], Vardan Galstyan [1] and Elisabetta Comini [1,*]

1SENSOR Lab, Department of Information Engineering, University of Brescia, 25133 Brescia, Italy

2Department of Physics and Electronics, Faculty of Science, University of Kelaniya, Kelaniya 11600, Sri Lanka

3Postgraduate Institute of Science, University of Peradeniya, Peradeniya 20400, Sri Lanka

4Department of Physics, Faculty of Science, University of Peradeniya, Peradeniya 20400, Sri Lanka

5Laboratory of Atomic Structures and Defects in Advanced Materials, National Institute of Materials Physics, Atomistilor str. 405 A, 077125 Magurele, Romania

6Research and International Affairs, Sri Lanka Technological Campus, Padukka 10500, Sri Lanka

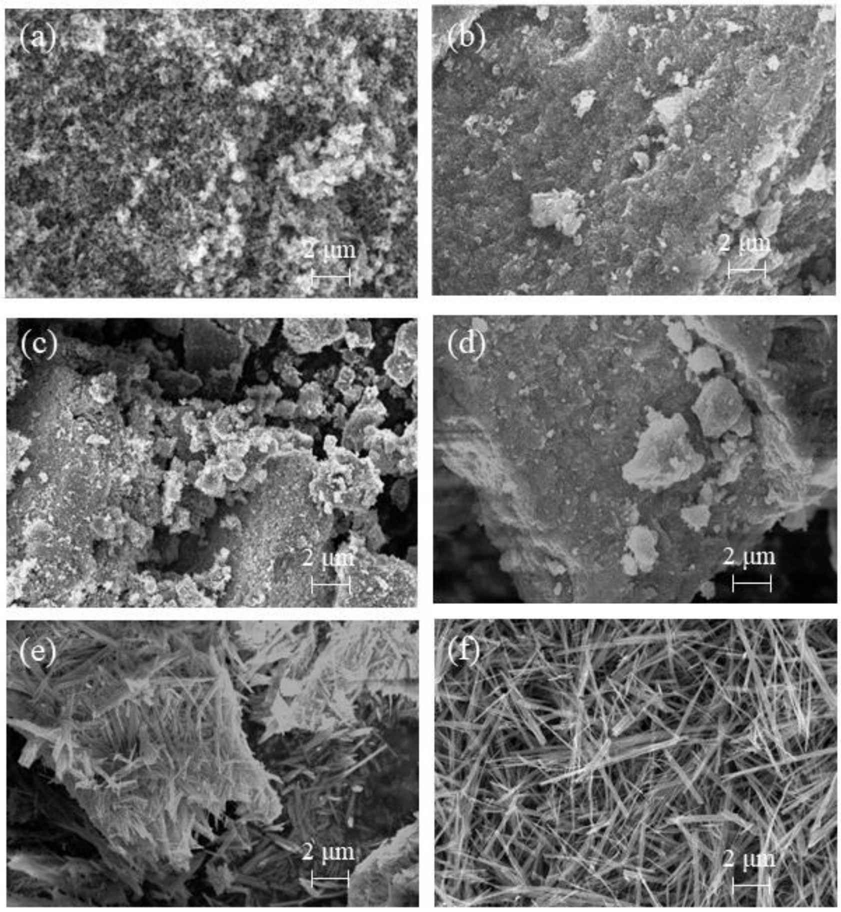

Figure S1. FE-SEM images of the obtained morphology at (a) starting anatase TiO_2 powder, (b) hydrothermal treatment at 120 °C, (c) hydrothermal treatment at 135°C, (d) hydrothermal treatment at 125 °C, (e) hydrothermal treatment at 175 °C, (f) hydrothermal treatment at 200 °C.

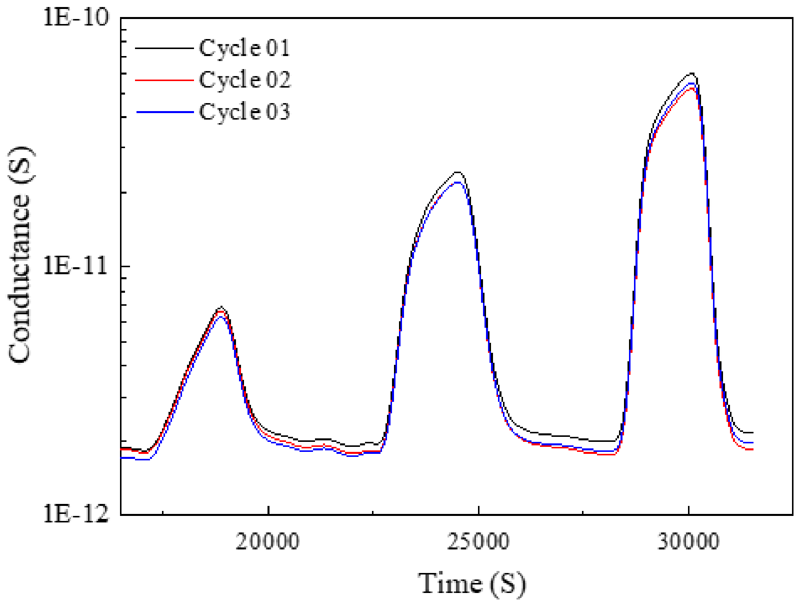

Figure S2. Reproducibility study of TiO_2-B nanobelts sensor toward 50 ppm C_3H_6O at 150 °C at 40 RH%.

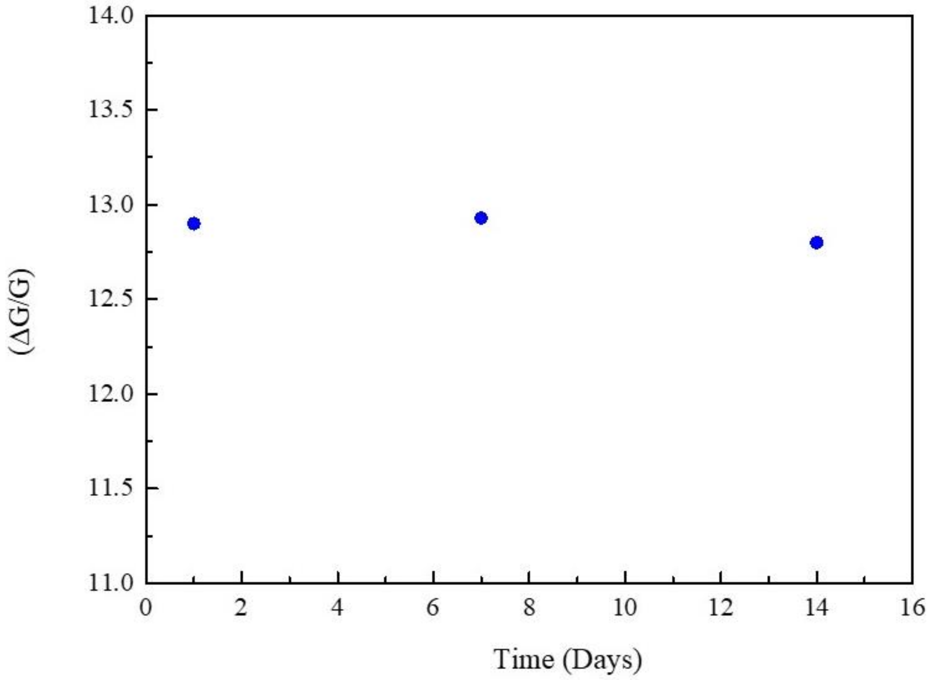

Figure S3. Stability study of TiO_2-B nanobelts sensor toward 50 ppm C_3H_6O at 150 °C in 40 RH% air for a tested two-week period.

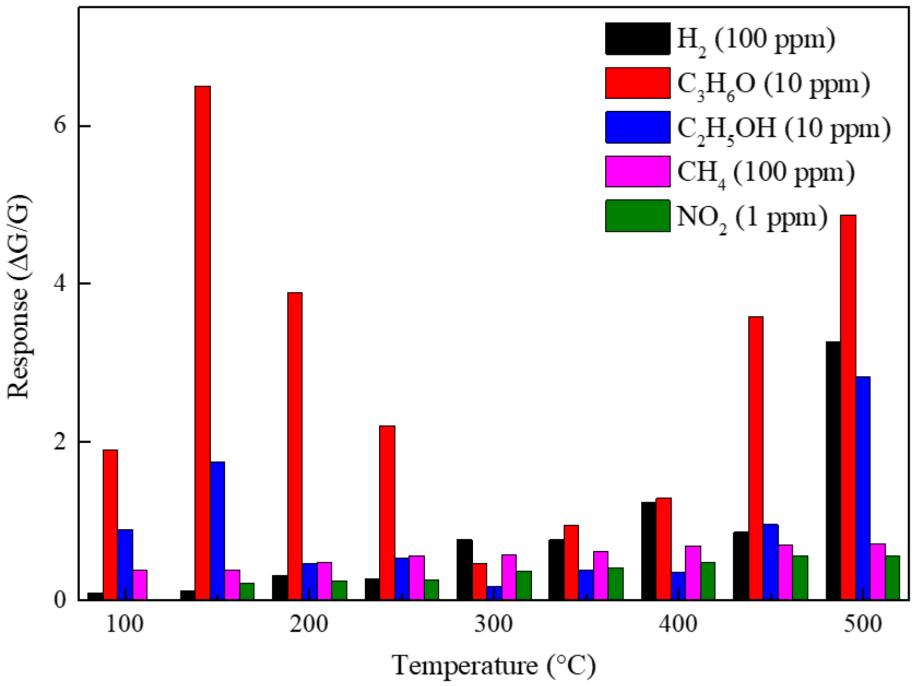

Figure S4. Response values of the sensor towards 500, 100, 10, 10, and 10 ppm of CH_4, H_2, NO_2, C_2H_5OH, and C_3H_5OH.
